# Tacrolimus and mycophenolate mofetil in corticosteroid-resistant hepatitis secondary to tislelizumab: a case report

**DOI:** 10.3389/fonc.2025.1385794

**Published:** 2025-02-03

**Authors:** Chang Jiang, Shanxian Guo

**Affiliations:** Department of Thoracic Oncology, Jiangxi Cancer Hospital & Institute, Jiangxi Clinical Research Center for Cancer, The second Affiliated Hospital of Nanchang Medical College, Nanchang, China

**Keywords:** immune-related hepatitis, tacrolimus, mycophenolate mofetil, corticosteroid, tislelizumab

## Abstract

Tislelizumab is a monoclonal antibody with high binding affinity for programmed death-1 (PD-1) receptors. In patients with extensive-stage small-cell lung cancer (ES-SCLC), the first-line use of tislelizumab combined with chemotherapy has shown significant efficacy. However, with the widespread use of PD-1 inhibitors, there are increasing reports of immune-related adverse events (irAEs) in clinical practice, with immune-related hepatitis (IRH) being particularly common. This article reports a case of an ES-SCLC patient (cT3N3M0 cStage IIIB) who developed corticosteroid-resistant hepatitis and recovered through dual immunosuppressant therapy. The patient was a 67-year-old male, diagnosed with ES-SCLC, who received a combination therapy of etoposide, cisplatin, and tislelizumab. Three weeks after the fourth treatment cycle, the patient experienced symptoms, such as decreased appetite, itching, yellow urine, and jaundice, and was diagnosed with IRH, manifested as “Grade 3 total bilirubin increase,” “Grade 3 alanine transaminase increase,” and “Grade 3 aspartate transaminase increase.” Despite intravenous injection of methylprednisolone (MP) 100 mg/day (2 mg/kg) and oral administration of mycophenolate mofetil (MMF) 1 g twice daily, liver function continued to be impaired. In this context, tacrolimus (TAC) (5 mg, twice daily) was added to the therapy, and the IRH level was reduced from Grade 3 to normal. Subsequently, TAC and MMF were gradually reduced and eventually discontinued. Unfortunately, after discontinuing immunosuppressants, IRH recurred. Although the patient still responded to TAC combined with MMF, liver function recovery took a longer time. Due to persistent liver dysfunction, the patient failed to receive second-line chemotherapy and ultimately passed away due to disease progression. Through this case, we hope to emphasize the importance of reasonably extending the use of immunosuppressants to avoid the recurrence of IRH and reduce the premature discontinuation of immunosuppressants. Besides, when tumor progression and IRH recurrence occur simultaneously, providing effective immunosuppressive therapy and reasonably arranging systemic anti-tumor therapy may bring clinical benefits to patients.

## Introduction

The emergence of immune checkpoint inhibitors (ICIs) has revolutionized the treatment landscape for patients with advanced solid malignancies demonstrating significant clinical benefits (1). However, the activation of T cells by ICIs can also lead to attacks on non-tumor normal tissues resulting in organ toxicity and immune-related adverse effects (irAEs), including immune-related hepatitis (IRH) (2). Most cases of IRH are mild to moderate, and interrupting ICI treatment and using corticosteroids can effectively control them (3). However, in a small number of patients with Grades 3 and 4 liver injury, corticosteroid alone may not be sufficient, and additional immunosuppressants, such as mycophenolate mofetil (MMF) or tacrolimus (TAC), are required (4, 5).

Tislelizumab is a human IgG4 monoclonal antibody developed by BeiGene Ltd. that binds to and blocks the programmed cell death-1 (PD-1) receptor expressed on activated immune cells, including T lymphocytes (6). Tislelizumab can enhance anti-cancer immune activity by blocking the binding of PD-1 to its ligand. Two early studies have shown that tislelizumab monotherapy has anti-tumor activity in patients with advanced refractory solid tumors (7, 8). The combination of tislelizumab and platinum-based chemotherapy as first-line treatment for advanced small cell lung cancer (SCLC) and non-small cell lung cancer (NSCLC) exhibited robust responses in a phase 2 study (9). In the RATIONALE-312 study, the incidence of IRH was 1.3%, and only one patient in the tislelizumab group (n = 227) reported ≥Grade 3 IRH (10). Here, we report a case of a Grade 3 IRH patient who was resistant to corticosteroid treatment and gradually recovered liver function after receiving MMF and TAC. Unfortunately, after discontinuing immunosuppressants, IRH recurred. Although the patient still responded to MMF and TAC, it took a longer time to improve liver function. During the treatment of hepatotoxicity, the patient was unable to receive systemic anti-tumor therapy and ultimately passed away due to disease progression.

## Case presentation

**Figure 1:**
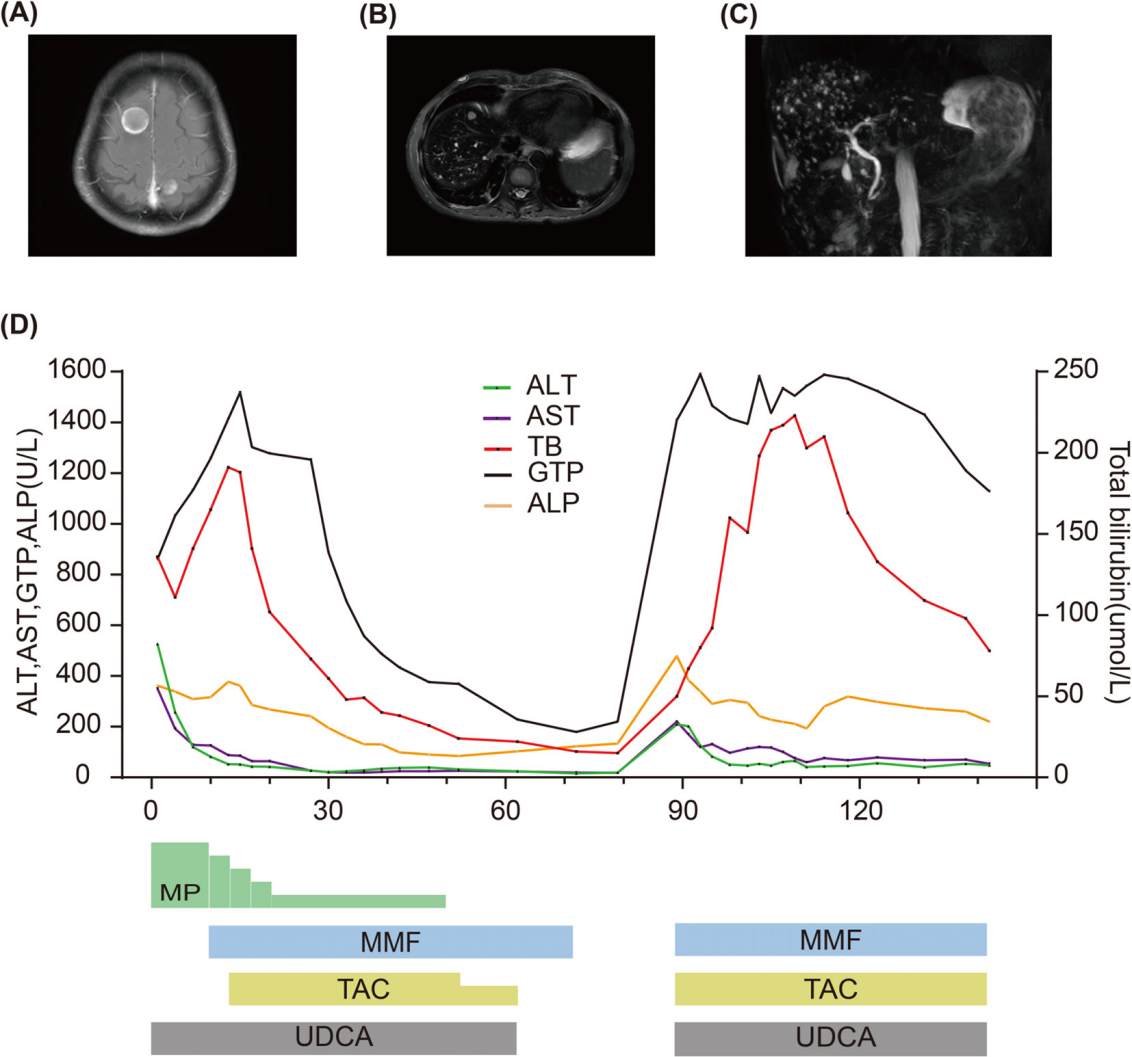
**(A)** MRI of the brain shows brain metastases. **(B)** MRI of the abdomen shows liver metastatic lesions. **(C)** MRCP performed when TB level was re-increased showing normal biliary tract. **(D)** Summary of clinical course and biochemical examinations.

A 67-year-old man with extensive-stage small-cell lung cancer (ES-SCLC) (cT3N3M0 cStage IIIB) received etoposide plus cisplatin in combination with tislelizumab (200 mg). Three weeks after completing the fourth treatment cycle, the patient started experiencing symptoms such as decreased appetite, itching, yellow urine, and jaundice. Liver function tests were conducted revealing the following results: total bilirubin (TB): 136 μmol/L (normal range: 0–26 μmol/L), alanine transaminase (ALT): 526 U/L (normal range: 0–50 U/L), aspartate transaminase (AST): 350 U/L (normal range: 0–40 U/L), alkaline phosphatase (ALP): 362 U/L (normal range: 45–125 U/L), and γ-glutamyl transpeptidase (GTP): 264 U/L (normal range: 10–60 U/L) (**Figure 1D**). According to the Common Terminology Criteria for Adverse Events Version 5.0, the patient was diagnosed with “Grade 3 total bilirubin increase,” “Grade 3 alanine transaminase increase,” and “Grade 3 aspartate transaminase increase.” Coagulation function and hemogram were normal. Chest CT showed near-complete resolution of lung lesions. Abdominal CT and ultrasound did not indicate liver metastasis or abnormalities in the hepatobiliary system. Anti-nuclear antibodies and anti-smooth muscle actin antibodies were negative. Tests for hepatitis B, hepatitis C virus, and HIV were also negative. The necessity of liver biopsy remains controversial in cases of suspected immune-related liver injury (11), and the patient declined this procedure, so no liver biopsy was conducted. Given the patient’s treatment history and clinical presentation, it was strongly suspected that liver dysfunction was related to immunotherapy. Therefore, the patient was diagnosed with IRH, and the PD-1 inhibitor tislelizumab was interrupted. The patient continued to receive ursodeoxycholic acid (UDCA) and was administered intravenous pulse methylprednisolone (MP) at a dose of 100 mg (2 mg/kg) for 3 days. Subsequently, the patient’s TB level and liver enzyme value decreased. Continuing the use of MP for 3 days resulted in a decrease in liver enzymes, but TB level increased. Although liver enzymes continued to decrease over the next 3 days, the persistent increase in TB led us to decide to add MMF at 1 g twice daily. However, we did not observe any improvement in TB level 3 days later. After starting oral MMF, TAC (5 mg) was added twice daily 4 days later. TB level gradually decreased by the 14th day of admission. Meanwhile, due to the patient’s resistance to corticosteroid, the MP dose was gradually tapered and eventually discontinued on the 50th day of admission. ALT and AST levels returned to normal by the 28th day of admission. The patient was discharged 39 days after hospitalization. Liver function was regularly followed up post-discharge. TB level returned to normal on the 52nd day since the initial detection of liver dysfunction. Then, the dose of TAC was decreased to 3 mg twice daily and ultimately discontinued on the 62nd day due to normalization of TB, ALT, and AST levels. MMF was then discontinued 10 days after stopping TAC (on the 72nd day).

The patient was readmitted on the 78th day due to dizziness, headache, and left supraclavicular lymph node enlargement. Pulmonary imaging examination revealed stable disease, and magnetic resonance imaging (MRI) detected brain metastasis (**Figure 1A**). An ultrasound demonstrated enlargement of the left supraclavicular lymph nodes. Liver function tests showed that TB, ALT, AST, and ALP were all within normal ranges (on the 79th day). The patient underwent a lymph node biopsy, and pathological results confirmed SCLC. A plan was devised to administer radiotherapy combined with second-line chemotherapy to the brain and left supraclavicular lymph nodes. However, before treatment commenced, the patient experienced nausea, yellow urine, jaundice, and liver dysfunction. Liver function analysis indicated abnormal TB at 50 μmol/L, ALT at 209 U/L, AST at 220 U/L, ALP at 479 U/L, and GTP at 1,409 U/L (on the 89th day). After the initial detection of liver dysfunction, the patient did not take any other medications except for UDCA, MP, and immunosuppressants. Based on the previous treatment history, the patient was diagnosed with IRH relapse. The treatment was restarted with 1 g of MMF twice daily, 5 mg of TAC twice daily, and UDCA for liver dysfunction. The patient underwent radiotherapy for the brain and left supraclavicular lymph nodes but could not receive chemotherapy owing to abnormal liver function. Following immunosuppressive therapy, the patient’s liver enzymes gradually decreased, but TB significantly increased. An abdominal MRI revealed multiple small metastatic liver lesions, with the largest lesion diameter being approximately 1 centimeter. Magnetic resonance cholangiopancreatography (MRCP) did not show any signs of biliary tract obstruction on the 93rd day (**Figures 1B, C**). After 28 days of oral administration of MMF and TAC, a gradual improvement in TB was observed. However, due to liver dysfunction and an Eastern Cooperative Oncology Group (ECOG) performance status of 3, the patient failed to receive second-line systematic chemotherapy. The patient required discharge on the 39th day of hospitalization (on the 107th day). Follow-up liver function tests showed a gradual recovery of TB. Due to the inability to receive systemic anti-tumor treatment, the patient eventually succumbed to disease progression.

## Discussion

ICIs have revolutionized the treatment of various solid tumors demonstrating remarkable clinical benefits (12). However, with the widespread application of these innovative therapies, the incidence of irAEs in clinical practice has gradually increased, with IRH being the most common type. The incidence of IRH caused by PD-1 inhibitor alone was approximately 1%–4%, while the incidence rate can rise to as high as 33% in patients receiving PD-1 inhibitors combined with CTLA-4 inhibitors (10, 13, 14). IRH is typically defined as an increase of at least three times in liver enzyme levels (15, 16). Its typical manifestations include elevated ALT and AST levels, which may or may not be accompanied by an increase in bilirubin (17). The onset of IRH usually occurs within 1–3 months, but it can also arise at any time. Notably, the management of IRH lacks strong randomized evidence, and existing treatment recommendations are primarily based on expert consensus from the European Society for Medical Oncology, the American Society of Clinical Oncology, the Society for Immunotherapy of Cancer, and the National Comprehensive Cancer Network (15, 16, 18). Generally, IRH associated with liver enzyme abnormalities may spontaneously resolve after discontinuing ICI treatment. Some patients require corticosteroid therapy to control IRH, while a small number of IRH patients who are resistant to corticosteroids necessitate the addition of immunosuppressants such as MMF, TAC, and cyclosporine. To date, the incidence rate of corticosteroid-resistant IRH is not well defined, and the evidence for its diagnosis and treatment is relatively limited. According to previous retrospective studies, approximately 23%–48% of IRH patients require additional use of immunosuppressants (19, 20). There is debate in clinical practice regarding the necessity of liver biopsy as an auxiliary tool for diagnosing corticosteroid-resistant IRH, but most guidelines recommend considering the need for liver biopsy based on specific clinical circumstances (15, 16, 18).

Herein, we report a case of corticosteroid-resistant Grade 3 IRH induced by tislelizumab. The patient developed IRH on the 15th week after starting tislelizumab treatment. The main clinical symptoms included decreased appetite, yellow urine, and jaundice. In addition to clinical manifestations, laboratory abnormalities associated with ICI included “Grade 3 total bilirubin increase,” “Grade 3 alanine aminotransferase increase,” and “Grade 3 aspartate aminotransferase increase.” According to a recent systematic review, approximately half of Grades 3–4 IRH patients achieved remission without receiving corticosteroid treatment (11), but this situation did not apply to our case. Despite pulse therapy with intravenous MP at 2 mg/kg, there was a decreasing trend in liver enzyme levels, but TB continued to rise.

For patients who did not respond to first-line MP treatment, MMF was recommended as a second-line treatment option and has been successfully applied to some corticosteroid-resistant IRH patients (5). Although MMF has achieved partial success in some cases of IRH, some patients have not seen significant therapeutic effects after treatment with steroids and MMF. Given the presence of CD8+ T-lymphocyte infiltration in histopathology, immunosuppressive agents that specifically target T cells, such as cyclosporine, TAC, and anti-thymoglobulin, may be the preferred third-line treatment. These therapies have been successfully applied in several cases (21–23). In addition, tocilizumab (IL-6 receptor antagonist) (24) and plasma exchange (25) have also been successfully used in some cases of steroid-refractory IRH. There are reports that the anti-TNF inhibitor infliximab normalizes liver function in steroid-refractory IRH patients (26), but not all guidelines recommend the use of this type of drug in IRH due to its potential hepatotoxicity (15, 16, 18).

In this case, despite the addition of the immunosuppressive agent MMF, an increase in TB was still observed. Immune-related cholestatic hepatitis is typically characterized by elevated levels of bilirubin, ALP, and GTP, indicating resistance to corticosteroids and a poor prognosis (27). Abdominal ultrasound or MRCP plays a key role in excluding factors of biliary obstruction. The abdominal ultrasound examination of this patient did not show any signs of biliary tract obstruction. Due to the patient’s refusal, a liver biopsy could not be performed. In this context, administering the calcineurin inhibitor TAC resulted in a gradual return of TB to normal level. Later, we adjusted the dosage of TAC and ultimately discontinued MMF and TAC. Unfortunately, after stopping MMF and TAC, the patient’s TB and liver enzymes showed abnormalities again. Although abdominal MRI showed metastatic liver lesions, MRCP showed no signs of biliary tract obstruction, and the patient did not take any hepatotoxic substances, the possibility of IRH relapse remained the top priority. MMF and TAC were orally administered again, and an improvement in liver enzymes was observed, but TB began to decrease until 28 days of therapy. Chemotherapy was not administered during the treatment of liver dysfunction, and the patient died due to tumor progression.

The process of IRH treatment suggests that IRH exhibits significant clinical heterogeneity, and its management remains challenging due to poorly understood pathogenesis, difficult diagnosis, and serious clinical consequences. Currently, there is insufficient evidence to support a specific duration for the use of immunosuppressants. Ziogas reported that resolving corticosteroid-resistant IRH with effective immunosuppressants may take up to 3 months, and the patient did not experience a recurrence of IRH (28). Hence, 3 months may be a reasonable duration for the use of immunosuppressants in these cases. In our subsequent clinical practice, immunosuppressants were administered for 3 months in corticosteroid-resistant IRH patients, and no patients experienced a recurrence of IRH.

In conclusion, our IRH patient exhibited resistance to corticosteroids but responded well to dual immunosuppressive therapy with MMF and TAC. Given the recurrence of IRH after discontinuing immunosuppressants, prolonging the treatment time of immunosuppressants to stabilize liver function may help to obtain opportunities for anti-tumor treatment. In addition, when tumor progression and IRH recurrence occur simultaneously, timely anti-tumor treatment may bring clinical benefits to patients, in addition to using initially effective immunosuppressive therapy.

## Data Availability

The original contributions presented in the study are included in the article/supplementary material. Further inquiries can be directed to the corresponding author.
